# Peripheral gene interactions define interpretable clusters of core ASD genes in a network-based investigation of the omnigenic theory

**DOI:** 10.1038/s41540-022-00240-x

**Published:** 2022-08-10

**Authors:** Ábel Fóthi, Csaba Pintér, Péter Pollner, András Lőrincz

**Affiliations:** 1 Department of Artificial Intelligence, Faculty of Informatics, Eötvös Loránd University, Budapest, Hungary; 2 Institute of Enzymology, Research Centre for Natural Sciences, Budapest, Hungary; 3 Institute of Archaeogenomics, Research Centre for the Humanities, Budapest, Hungary; 4 MTA-ELTE Statistical and Biological Physics Research Group, Eötvös Loránd Research Network (ELKH), Department of Biological Physics, Eötvös University, Budapest, Hungary; 5 Health Services Management Training Centre, Semmelweis University, Budapest, Hungary

**Keywords:** Genetic interaction, Diseases

## Abstract

According to the recently proposed *omnigenic* theory, all expressed genes in a relevant tissue are contributing directly or indirectly to the manifestation of complex disorders such as autism. Thus, holistic approaches can be complementary in studying genetics of these complex disorders to focusing on a limited number of candidate genes. Gene interaction networks can be used for holistic studies of the omnigenic nature of autism. We used Louvain clustering on tissue-specific gene interaction networks and their subgraphs exclusively containing autism-related genes to study the effects of peripheral gene interactions. We observed that the autism gene clusters are significantly weaker connected to each other and the peripheral genes in non-neuronal tissues than in brain-related tissues. The biological functions of the brain clusters correlated well with previous findings on autism, such as *synaptic signaling*, *regulation of DNA methylation*, or *regulation of lymphocyte activation*, however, on the other tissues they did not enrich as significantly. Furthermore, ASD subjects with disruptive mutations in specific gene clusters show phenotypical differences compared to other disruptive variants carrying ASD individuals. Our results strengthen the omnigenic theory and can advance our understanding of the genetic background of autism.

## Introduction

Autism spectrum disorder (ASD) is a complex neuropsychiatric disorder both from the point of view of behavior and genetics^1,2^. The diagnosis of the disorder is based on the results of behavioral tests of the core symptoms in social behavior, speech/communication, repetitive behavior, and restricted interests^3^. Furthermore, the identification of autism-related genes is at the forefront of research^4^. However, the concepts of both core behavioral symptoms and “autism genes” are recently challenged^5,6^. Autism is a high-complexity disorder, and the phenotypic and genetic heterogeneity could not be explained by a small number of factors, therefore holistic approaches seem advantageous^7,8^. The recently proposed omnigenic theory^9^ is a promising novel way to describe high-complexity disorders like autism from genetic background. The omnigenic theory divides genes into two main categories: core genes that have an identifiable direct impact on complex disorders, and peripheral genes which do not have a direct impact but are expressed in relevant tissues and affect the expressions of the core genes. Peripheral genes have an indirect influence on the disease by interacting with core genes and regulating them through peripheral gene interaction networks.

Although omnigenic theory has many supporters, its reception is not unequivocal. Wray et al.^10^ highlight the risk of putting too much emphasis on core genes. Another critique of the omnigenic theory concerns the strict distinction between core and peripheral genes^11^. In a commentary paper from Franke^12^, a gradual distinction of core and peripheral genes was suggested. Liu et al.^13^ have been trying to overcome the limitations of the original omnigenic model by giving a more precise definition of core genes and introducing a practical systematic approach to study the omnigenic hypothesis quantitatively. They defined a model, which aggregates *cis* and *trans* effects on the core genes to explain heritability. *Cis*-regulatory elements are on the same molecule as their target; hence they can directly regulate the core genes. The most prominent examples of *cis*-regulatory elements are transcription factor binding sites. Genetic variants in *cis*-regulatory elements can have a direct effect on the expression of core genes, and they can be defined by expression quantitative trait loci (eQTL)^14^ or predicted by machine learning approaches, such as Expecto^15^. On the other hand, peripheral genes expose their influence through *trans* effects, and in fact, a significant portion of gene expression heritability is due to *trans* variants^13,16^. In turn, the systematic evaluation of the omnigenic theory requires the application of a quantitative description of gene–gene interaction between the peripheral and core genes. Gene interaction networks, especially when gene co-expression data is also integrated, seem appropriate for this purpose. Although *trans* effects and gene interactions are important factors of the omnigenic model, this aspect of the theory still has a long way to evolve in understanding the interactions of peripheral genes and core genes in order to analyze the genetic background of complex disorders.

GIANT^17^ (Genome-scale Integrated Analysis of gene Networks in Tissues)—created by Greene et al.—integrated many gene co-expression and functionality databases into tissue-specific gene interaction networks. Edges of the networks are weighted according to the tissue-specific posterior probabilities of the functional relationships between the pairs of genes. A brain-specific GIANT network was subsequently used to analyze ASD genes by Krishnan et al.^18^. This work expanded the set of core ASD genes by machine learning on the gene interaction network. They also demonstrated that Louvain modularity^19^ is an effective way to find clusters of this extended set of core ASD genes that can be described with distinct biological processes. They could define nine clusters: one for cellular and neuronal functions, three for signaling pathways, one for histone modification and chromatin remodeling, one for cell cycle regulation, one for enteric nervous system development, one for a whole assortment of perceptions, and one for circadian rhythm. However, their approach was based on the interaction between ~10% of the genes expressed in the brain, which is still a limited number of genes compared to an omnigenic model.

Empirical graphs such as social networks and gene interaction networks have common statistical properties as the small-world property, power-law degree distributions, and the property of community structure, in which the nodes form dense clusters, which are loosely connected^20^. The Louvain method efficiently detects these communities even in large networks. From a biological perspective, the members of a community on a gene interaction network tend to have stronger functional interactions with each other than with the members of other clusters^19^.

The motivation of the present study was two-fold. First, we aimed to quantitatively test whether the tissue-specific gene interaction network of the brain, a tissue with neurodevelopment relevance, is more suitable for the implementation of the omnigenic model to autism rather than other networks without neurodevelopmental roles (e.g., tissues from kidney, lung). We hypothesized that the functional clusters of the autism genes are embedded into larger clusters of brain-specific gene interactions. Second, we wanted to exploit the community structure of gene interaction network of the brain to find clusters of autism genes with enriched biological functionality and connections to autism phenotypes.

## Results

### Autism genes are central nodes in brain-related gene interaction networks

We analyzed the properties of autism-related genes in 144 tissue-specific gene interaction networks from the GIANT database. All networks have a similar number of nodes between 25,689 and 25,825, however, the number of their edges shows a high discrepancy: it ranges from 30,879,035 to 282,977,319.

Putative core genes were selected based on the Simons Foundation Autism Research Initiative (SFARI) Gene database. SFARI genes with gene scores 5 and 6 were excluded, thus the set of putative core genes contains 756 genes tied to autism based on empirical evidence. We hypothesize that the subnetwork of the SFARI genes on the GIANT network reveals the core subgraph of autism. However, it does not imply that all genes of the core subgraph are genuine core genes. Since different numbers of SFARI genes are connected to each other in each tissue-specific network, the sizes of core subgraphs differ from tissue to tissue.

A common measure of the node’s importance is node strength^21^, which can highlight the central nodes in a weighted network. Strength is proportional to node degree and edge weight, therefore in GIANT, a set of genes with multiple strong edges are more probably to form a functional cluster than genes that are connected by weaker edges.

To quantify their importance, we measured the node strengths of SFARI genes in the corresponding full graphs. The 144 available tissues were divided into two groups: 37 brain-related and 107 non-brain-related tissues (Supplementary Data 1). In 16 from the non-brain-related tissues and in 15 from the brain-related tissues SFARI genes have significantly higher strength than other genes (*p*: 2.303 × 10^−4^, Chi-squared test), thus brain-related tissues are 2.7 times enriched among the tissues, in which SFARI genes are central.

For further investigations, three tissues were selected from the GIANT database. Brain is the most relevant tissue for neuropsychiatric disorders and SFARI genes are central in its network. For comparison, two other networks were selected with edge numbers of the same magnitude as in the brain: the kidney and the lung networks (Table 1).

**Table 1:** Network properties of the tissue-specific full and core subnetworks.

Tissue	Nodes		Edges		Average node strength		
	Full	Core	Full	Core	Full	Core*	Core
Brain	25.6k	745	42 m	43.7k	375.6	417.4	17.66
Kidney	25.6k	720	62 m	16.6k	509	319.7	6.97
Lung	25.6k	684	60 m	15.3k	495.9	307.7	6.88

Number of nodes, edges, and the average node strengths are listed for the tissue-specific (Brain, Kidney, Lung) GIANT networks (Full), and their ASD core subnetworks (Core). “Core*” indicates the average node strength of core genes while all edges are kept from the full network.

Comparing full networks of gene interactions, kidney and lung networks have 48% and 43% more edges, respectively, than the full network from the brain tissue. However, on core subgraphs this changes in favor of the subnetwork of the brain, which consists of 2.63 and 2.86 times more edges than the subgraph from the kidney and from the lung (Table 1).

**Fig. 1:**
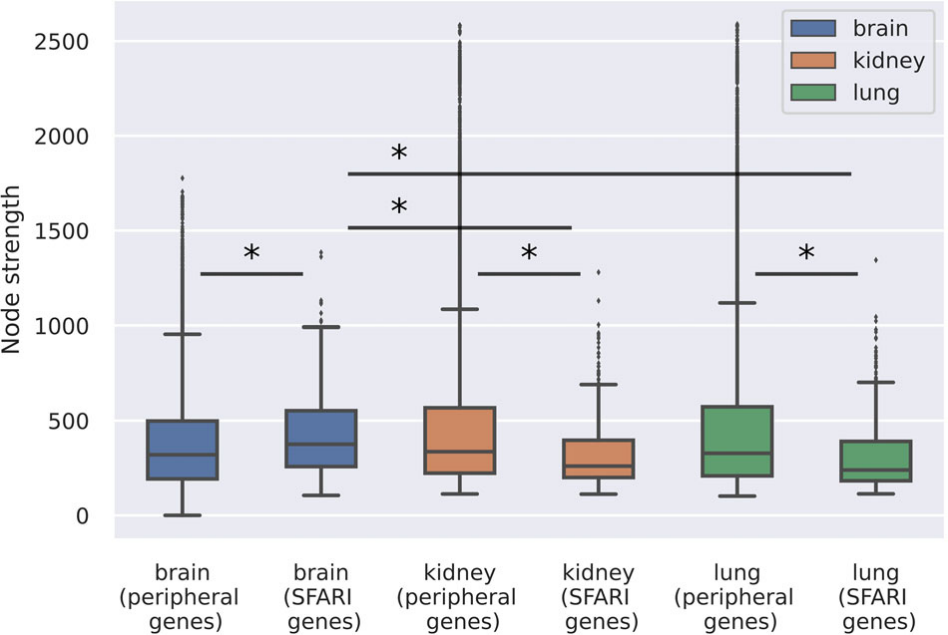
Core ASD genes have stronger connections in relevant tissues. The box plots show the node strengths of the peripheral genes and the SFARI genes for brain, kidney, and lung networks (centerline, median; box limits, upper and lower quartiles; whiskers, 1.5x interquartile range; diamonds, outliers). Statistical test: Mann–Whitney U test, significant differences are marked by *(*p* < 0.05).

Furthermore, the central role of ASD genes in the relevant brain tissue is reinforced by the average node strengths of the core subnetworks: 17.66 for the brain compared to 6.97 and 6.88 for the kidney and the lung, respectively. In fact, core genes are more scattered with weaker connections on ASD-irrelevant tissues, while they become central elements with stronger connections in the brain network. This tendency is also observable in the “Core*” networks (where core node strengths are calculated on the full network). Node strengths of the SFARI genes were compared to the node strength of the peripheral genes in all available tissue (*n* = 144), and among them, three representative tissues were shown on the box plot diagram (centerline, median; box limits, upper and lower quartiles; whiskers, 1.5x interquartile range; diamonds, outliers) (Fig. 1). Node strengths of the SFARI genes on the networks of the kidney (319.712, 95% Confidence Interval (CI) [307.511, 331.913]) and lung (307.723, 95% CI [294.828, 320.618]) are significantly lower (*p*: 2.47 × 10^−26^ on kidney, *p*: 3.033 × 10^−29^ on lung, Mann–Whitney U-test) than for the peripheral genes (kidney: 514.627, 95% CI [508.791, 520.463]; lung: 501.501, 95% CI [495.731, 507.272]). Furthermore, the node strengths of the SFARI genes in the brain (417.404, 95% CI [402.288, 432.52]) are higher than the values for either the SFARI genes in the irrelevant tissues (*p*: 1.68 × 10^−23^, 4.146 × 10^−31^ compared to the kidney and the lung, respectively, Mann–Whitney U test) or the peripheral brain genes (374.352, 95% CI [371.341, 377.363], *p*: 1.079 × 10^−13^, Mann–Whitney U test).

### The highest core-periphery similarity is in the brain

According to the omnigenic hypothesis, we should consider the relationships not only between the core genes but also the influences from the peripheral genes.

Peripheral influence is propagated through the gene interactions that connect the peripheral genes to each other and to the core genes. These edges are part of the full network, but they are ignored in the core subnetwork. It also means that the full network represents both the core subnetwork and the peripheral influence on them. Community detection on the GIANT networks finds functional clusters, in which genes have a higher probability to be in a functional relationship with each other than with genes from other clusters. When the full network has a different community structure than the core subnetwork, the peripheral influence weakens the core clusters. On the other hand, interrelated full and core communities are strengthened by peripheral influence and these clusters of autism genes are core clusters even in the omnigenic framework.

**Fig. 2:**
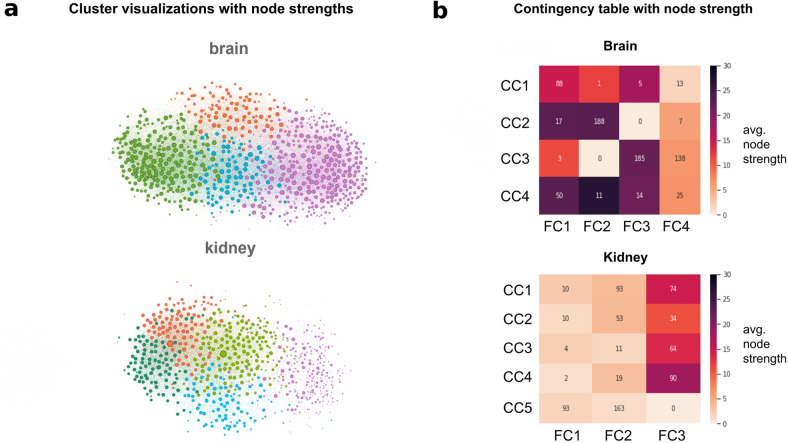
SFARI genes define core clusters. **a** Visualization of the core gene clusters. Colors correspond to the different clusters; nodes are sized based on their node strengths. **b** Contingency tables that provide the basis for Cramers’ V calculation. Numbers in cells of the table indicate the number of intersecting genes between clusters of the subgraph (CC, rows) and the full network (FC, columns). Colormaps are based on each subcluster’s average node strength to further demonstrate the centrality differences between the two tissues.

Community detection divided both the full and the core networks into smaller clusters. The clusterings of two representative core subnetworks (from brain and kidney) are visualized in Fig. 2a, where the size of the vertices corresponds to their node strength. Clusters found by the Louvain method are marked with different colors. This provides visualization for our observation that in tissues with closer relevance to autism, SFARI genes have substantially stronger connections than in less relevant tissues.

Community detection on the full brain network detected 4 large clusters with 10939, 4748, 3720, and 6267 genes and 3 small clusters with 6, 5, and 4 genes. The core subnetwork was divided into 4 clusters having 326, 212, 107, and 100. We compared the two sets of clusters by taking their intersections. Intersections of the core clusters and the full clusters define the subclusters and they are shown in contingency tables. The rows correspond to the clusters on the core subgraphs, the columns correspond to the clustering on the full networks and the cells of the contingency tables show their intersections. With this contingency table, each core cluster is further subdivided by considering the peripheral influences. Figure 2b shows the contingency tables with a colormap that is colored based on the average node strengths of the subclusters. On the brain core subgraph, the 4 core clusters were further divided into 16 subclusters by intersecting with the 4 clusters of the full brain network. On the kidney core subgraph, there are 5 clusters, while the kidney full network has 3 clusters, this gives us a contingency table with 5 rows and 3 columns.

Resulting contingency tables were used for testing, whether the clusters of the autism-related genes are strengthened by the peripheral interactions of the full network or not. If there is a relationship between the community structures of the full network and the core network, we expect to have the clusters in the core network to be embedded in their corresponding clusters of the full network. In contrast, when the nodes of genes of autism are independently distributed over the full network of tissue-related gene expression, a homogeneous overlap between core clusters and full clusters is expected.

The hypothesis is tested by investigating the distribution of the genes among the subclusters. Even distribution would indicate that the peripheral influence is not correlating with the core connections between the autism-related genes in the tissue. On the other hand, each core cluster may have its community on the full network, i.e., the numbers in the contingency table can be concentrated into single cells in each row and each column. In this case, the autism genes are expressed in the tissue according to the peripheral interactions.

Proceeding from these expectations, we introduce a core-periphery similarity measure, which quantitatively indicates the interrelation between the core and full network communities in a tissue. This measure should be high for strongly related core-periphery community structures, and it should take lower values for tissues with weak correlation between the core and peripheral gene interactions. Furthermore, as the sampled subgraphs should represent the community structure of the full network, these subgraphs are expected to have a relatively high core-periphery similarity. Because of technical considerations as the contingency table may have different dimensions for different tissues, Cramer’s V statistics were used as a quantitative measure.

**Fig. 3:**
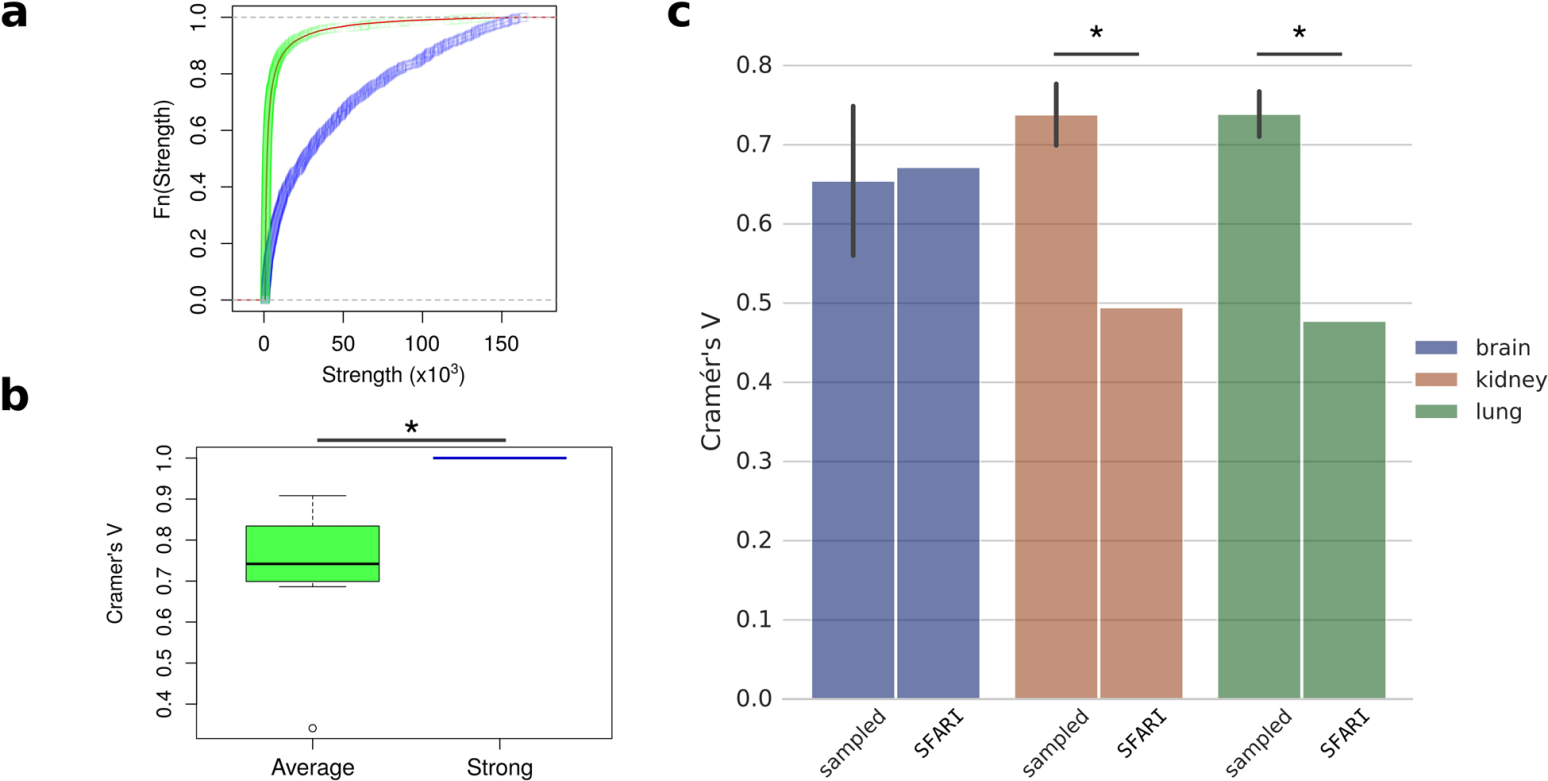
Core-periphery community similarity measurements. **a** Node strength cumulative distribution functions were calculated on all nodes (red line), sampled nodes with average node strength distribution (green), and sampled strong nodes (blue) of the LFR benchmark network. **b** Bar plots show the Cramer’s V values of replicated selection (*n* = 10) of nodes with average (green) and strong nodes (blue) of the LFR network (centerline, median; box limits, upper and lower quartiles; whiskers, 1.5x interquartile range; circles, outliers). **c** Cramer’s V value of sampled and core (SFARI) subnetworks of three tissues are presented: brain (blue), kidney (red), and lung (green). ‘Sampled’ indicates repeatedly sampled subgraphs (*n* = 10), with error bars showing standard deviation. Statistical test: **b**, *t*-test; **c** one sample *t*-test; significant differences are marked by *(*p* < 0.05).

In order to test this approach, we used Lancichinetti–Fortunato–Radicchi (LFR) benchmark algorithm and generated a network with 25,000 nodes and 4 communities (see “Methods”). 745 nodes were repeatedly selected and their subnetworks were constructed similarly to the core subnetwork of SFARI genes. Two groups of nodes were selected: a group of nodes with average node strength and another with high node strength. The cumulative distribution functions of the LFR network and the two selected subnetworks are depicted in Fig. 3a. Nodes with high strength kept the community structure of the benchmark network. They clustered accordingly to the priori known communities of the LFR network, hence their Cramer’s V values are 1. On the other hand, peripheral nodes with average node strength form communities with less similar structures, and therefore their Cramer’s V values are significantly smaller (mean: 0.738; *p*: 6.223 × 10^−4^, df: 9, 95% CI [−0.377, −0.146], two-sided, two-sample *t*-test) (Fig. 3b).

After validating that Cramer’s V value can measure the core-periphery similarity, the GIANT networks were also tested. Subgraphs were sampled from the full networks by strength-based node selection as a basis for comparison. The sampled subgraphs contained as many nodes as the number of core genes in the given tissue. We speculated that the sampled subgraphs represent well the community structures of the full network. Therefore, we expect a moderate concentration of numbers in their contingency tables. Cramer’s V values of the sampled subnetworks of irrelevant tissues have a mean value of 0.738 with a 95% CI [0.709, 0.767] on kidney, and a mean value of 0.739 with 95% CI [0.717, 0.76] on lung. These values are significantly higher than the Cramer’s V values of the ASD core subnetworks on the same tissues (0.48 for lung and 0.49 for kidney, *p*: 1.551 × 10^−08^, df: 9; *p*: 5.238 × 10^−10^, df: 9, respectively, one sample *t*-test) (Fig. 3c). On the other hand, comparison of sampled subnetworks with the core subnetwork in the relevant brain tissue results in very similar dependency values (0.654, 95% CI [0.583, 0.726] and 0.672, respectively, *p*: 0.596, df: 9, one sample *t*-test). It means that the clusters of the sampled subnetwork and the core clusters are similarly aligned to the communities of the brain full network. These results imply that peripheral genes form similar communities as the core autism genes in the brain, while genes in the kidney and the lung form weakly related core and periphery community structures.

Comparison between the genes’ node strengths and their position in the contingency matrices shows that the brain network has strongly connected nodes in its largest subclusters, while in the kidney networks, the genes in the largest subclusters have relatively small node strengths (Fig. 2b). Furthermore, on the kidney, 4 of the top 5 subclusters with the highest node strengths are in the same cluster of the full network (FC3, 3rd column of the contingency table). It strengthens that the interaction between ASD genes does not align with the community structure of the full kidney network and the full clusters are organized around different sets of central nodes than the core clusters. In turn, in the brain, the subclusters with the highest node strengths are distributed more evenly among the full clusters in accordance with the higher core-periphery similarity.

### Functional analysis of the tissue-specific clusters

We used Gene Ontology enRIchment anaLysis and visuaLizAtion (GOrilla) tool^22^ to perform Gene Ontology (GO) analysis both on the core clusters and subclusters. On the network of tissues, enrichments were calculated from the enrichment of GO terms on the core clusters or the subclusters compared to the background set of all expressed core genes in the given tissue. We tested whether the brain-specific clusters of core genes could be functionally better described than the irrelevant clusters of the same genes or not.

Comparisons of the tissue-specific core clusters were performed from multiple perspectives: first by analyzing the number of GO enriched genes of the core clusters (according to the p-values of the GOrilla results). The number of enriched genes is higher for the brain (531) than for the kidney and the lung (482 and 508, respectively). It implies that SFARI genes form functionally more homogenous clusters in the brain than in the other two networks.

Secondly, we also compared the number of associated GO terms. Core clusters of the brain have 265 associated GO terms, while the kidney and the lung have 199 and 187, respectively. These results indicate that on the brain tissue more core genes are enriched, and more GO terms associated with them, than on the irrelevant tissues.

**Fig. 4:**
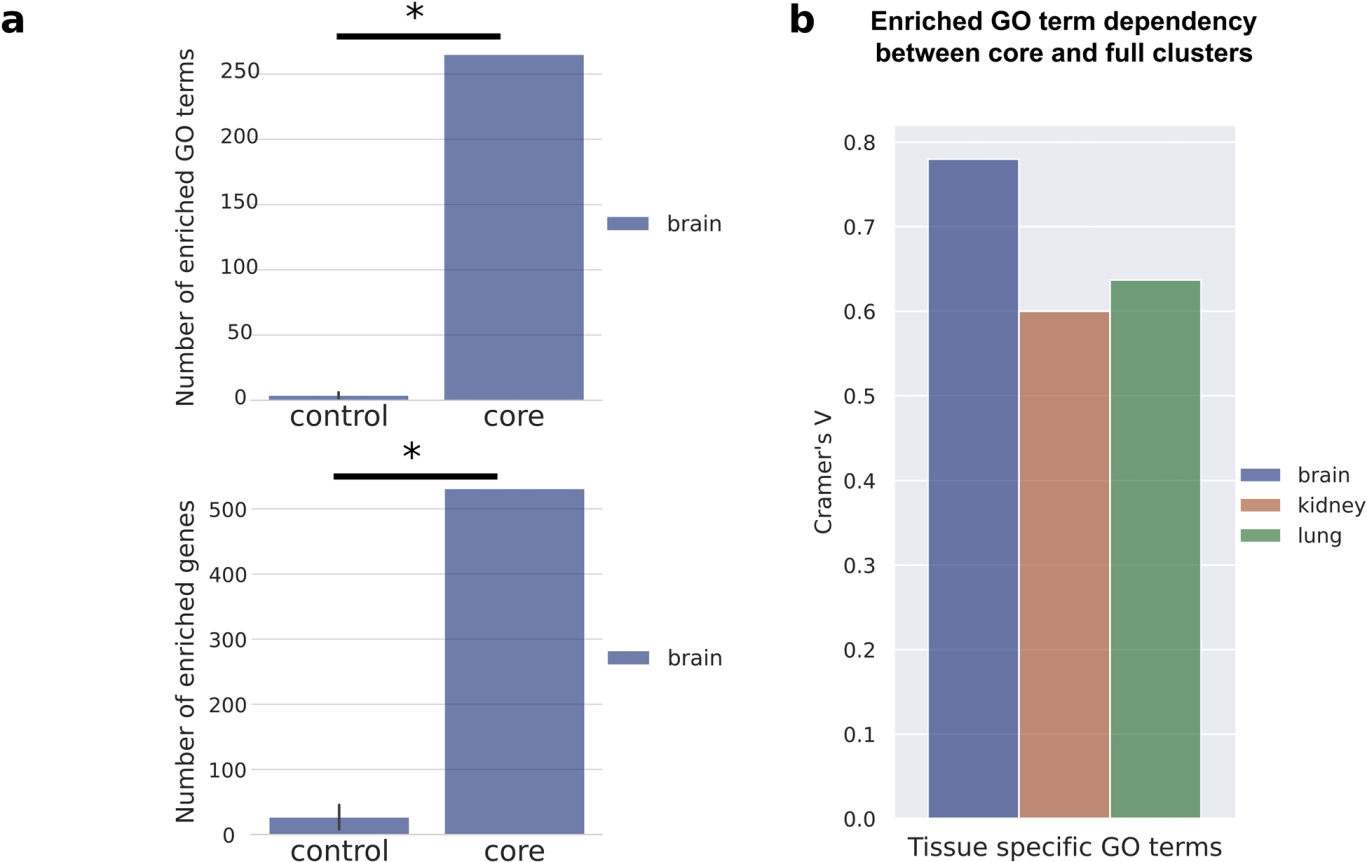
SFARI genes form functionally enriched clusters and subclusters on the brain network. **a** The bar plots show the number of enriched GO terms (upper panel) and the number of enriched genes (bottom panel). The control group consists of the size matching new clusters of the regrouped sets of SFARI genes, while the core shows the enrichment values on the original brain core clusters of SFARI genes. **b** The bar plots show the functional similarity between core clusters and full clusters of brain, kidney, and lung from the perspective of the number of enriched GO terms. Statistical test: **a**, one sample *t*-test, significant differences are marked by *(*p* < 0.05).

For further validation of the functional clusters, we regrouped the SFARI genes into size matching new clusters and tested whether the same terms will be enriched in these new sets of SFARI genes or not. Our results show that SFARI genes lose their functional homogeneity in these reorganized sets (Fig. 4a). In the regrouped clusters the mean of the enriched GO terms is 3.8 (95% CI [0.708, 6.892]) and they are associated to 26.6 genes on average (95% CI [0.679, 52.521]). Both are significantly lower than the values of the original clusters (*p*: 7.026 × 10^−7^, df: 9 for the number of GO terms (265) and *p*: 1.982 × 10^−9^, df: 9 for the number of genes (531), one sample *t*-test).

Finally, we investigated the dependency between the core clusters and the full networks. We calculated the Cramer’s V values on the distributions of GO terms in the tissue-specific contingency tables. The number of enriched GO terms showed stronger peripheral dependence in the brain (0.7801) than in the kidney (0.6003) and lung (0.6366) (Fig. 4b).

**Fig. 5:**
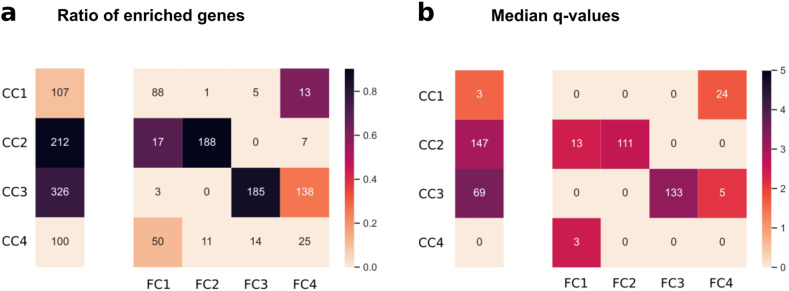
Functional enrichments show that subclusters on brain tissue keep their functional comprehensibility. The separate vertical vectors illustrate exclusively the core clusters, while the rows and columns of the contingency tables determine the intersections of core and full clusters, respectively. Numbers in the cells of the vectors and the tables represent **a** the number of genes and **b** the number of associated GO terms of core clusters and the subclusters., respectively. Colormaps show **a** the ratio of genes that are enriched (enriched/total genes in the cell) and **b** median of the –log(*q*) values associated with the enriched GO terms in the cell.

We also compared the functional interpretability of the core clusters and subclusters. Dividing sets of genes into smaller subsets has the possibility of diminishing efficiency in finding enriched biological processes because parallel to decreasing group sizes it could be increasingly difficult to identify homogeneous subgroups of genes. In order to test whether the clusters diminished their functional comprehensibility due to the subdividing, we compared the ratio of enriched genes, the number of associated GO terms, and the significance of these GO terms between core clusters and their derivatives, the subclusters. In order to control the high rate of type I errors arising from multiple hypothesis testing, we calculated corrected *p*-values (*q*-values) by using false discovery rate (FDR) controlling. For each core cluster and subcluster, we calculated the median –log(*q*) value to describe the strength of enrichment. Results were visualized similarly to the node strengths: the obtained values were represented by a single vector for the core clusters and a contingency table for the subclusters, both the ratio of enriched genes (Fig. 5a) and for the number of GO terms (Fig. 5b).

The ratio of enriched genes for the entire first core cluster is 0.1. If we look into the subdividings, out of the 4 potential subclusters only one is enriched significantly with 0.1 enrichment ratio. In the other three clusters, no significant enrichment is found. The number of associated GO terms for this core cluster is 3, while this increases to 24 for the enriched subcluster. The median –log(*q*) for the entire core cluster is 1.63 while 1.74 for the subcluster.

The second core cluster has two enriched subclusters. The entire core cluster has an enrichment ratio of 0.9 from 147 associated GO terms, while the two subclusters are similar, with enrichment ratios of 0.71 and 0.9 from 13 and 11 associated GO terms, respectively. The median –log(*q*) is 2.95 for the whole core cluster, and this value becomes slightly smaller on the subcluster level: 2.43 and 2.61, respectively.

The third core cluster also has two enriched subclusters, however, these are larger in the number of genes relative to the second core cluster. Compared to the core cluster with an enrichment ratio of 0.77, one of the subclusters improves this number to 0.86, despite having fewer genes (185 instead of 326), while the other one has a smaller ratio (0.26). The more enriched subcluster also has stronger significance (–log(*q*): 3.18 vs. 2.11), although both of these are weaker than the entire core cluster’s (–log(*q*): 3.46). The number of associated GO terms are 133 and 5 for the subclusters and 69 for the entire core cluster, respectively.

The last cluster has no enrichments. However, on the subcluster level, there is one, with an enrichment ratio of 0.12, median –log(*q*) of 2.49 from 3 associated GO terms.

In both metrics, subclusters kept the enrichment ratio of genes and the number of associated GO terms—instead of letting it spread across subclusters as it would be mathematically expected (without biological aspects). This preservation of biological meaning implies the effectiveness of the peripheral influence-based subdividing.

### Peripheral interactions of the brain network divide core gene clusters into functionally interpretable smaller clusters

Statistical analysis of the Gene Ontology Enrichment Analysis results suggests that peripheral influence on core genes enables biologically plausible subdividing. To strengthen this observation, we investigate the most prominent GO terms for each cluster and subcluster and whether they are previously connected to autism, or not.

**Fig. 6:**
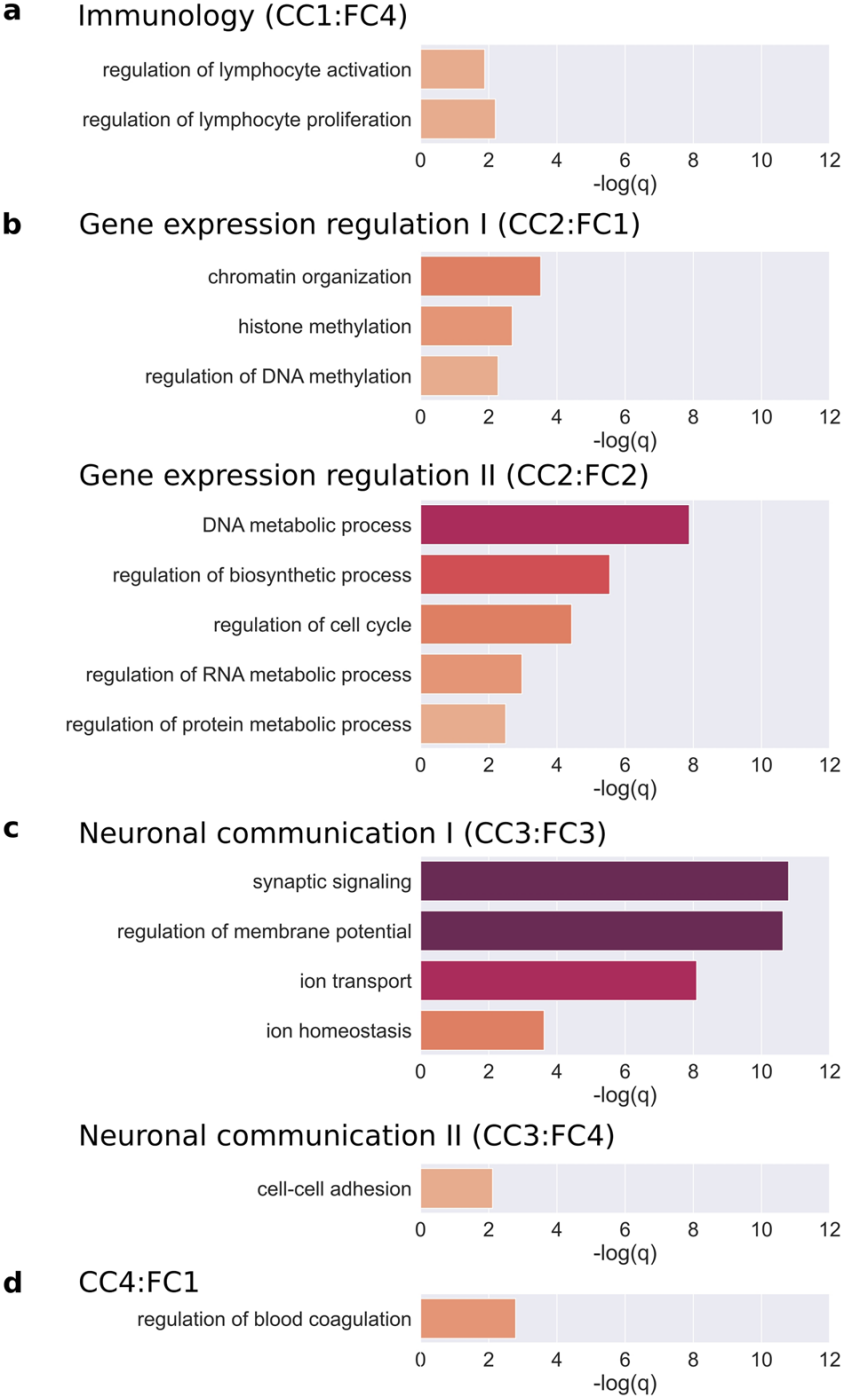
Subdividing by peripheral influences results in functionally interpretable subclusters of autism core genes. **a**–**d** −log(*q*-values) of the GO terms associated with the 6 comprehensible subclusters of the 4 core clusters.

The size of the first cluster (CC1) is relatively small (107 genes). GO terms for this group are not enriched as strongly as for the second and the third clusters, but they are still significant. The largest term is *regulation of intracellular signal transduction*, which includes 30 genes, among them important autism genes such as *mTOR*. However, the most significant processes are related to *immunology*. Peripheral interactions further subdivide this cluster first, to a general white blood regulatory subcluster with significant terms such as *regulation of lymphocyte activation* and *regulation of lymphocyte proliferation* (CC1:FC4), and second, a statistically insignificant (*q* > 0.05, but *p* < 0.001, hypergeometric test) regulatory subcluster related to *regulation of interferon gamma production* (CC1:FC1) (Fig. 6a).

The second main cluster (CC2) is larger than the first, it contains 212 genes. In this cluster, the processes include (1) protein modifications such as *histone modifications*, *protein ubiquitination,* and *peptidyl-lysine modification*, (2) *DNA metabolic processes* such as *DNA repair* and (3) *RNA metabolic processes*. The most significant process for the cluster is *chromatin organization* (*p*-value: 6.42 × 10^–14^ and *q*-value: 4.37 × 10^–10^, hypergeometric test). 75 core genes are involved in *chromatin organization*, of which 51 fall into this cluster (enrichment: 2.38). Its subprocess, *histone modification*, is also enriched strongly (33/48 genes, 2.41 enrichment). In addition, other genes of CC2 play a role in the *regulation of gene expression* (107/251 genes, 1.49 enrichment). In summary, in CC2 there are mainly genes that are able to regulate the level of gene products formed, either through gene expression or the necessary metabolic processes. Interestingly, this result also showed that the epigenetic regulators appeared in the same cluster as the regulators of metabolic processes. However, subdividing by the peripheral influences separated the genes into these two distinct sets of processes (Fig. 6b). The first subcluster (CC2:FC1) contains genes related to epigenetics as *chromatin organization*, *histone methylation*, and *regulation of DNA methylation*, while the genes in the second subcluster (CC2:FC2) have metabolic regulatory functions like *regulation of biosynthetic processes, regulation of RNA metabolic process, DNA metabolic process, regulation of protein metabolic process*. In addition, the genes related to the *regulation of cell cycle* are also members of this subcluster.

The third core cluster (CC3) consists of the most genes (326 genes). Based on biological processes, the most strongly enriched set of genes is related to *membrane depolarization* (15/15 genes, 2.3 enrichment). Furthermore, a large number of genes are related to *glutamatergic transmitters*, *calcium ion transporters*, and *regulators of synaptic vesicle clearance*. Based on these, this cluster mostly contains genes that are responsible for neural communication. At the same time, in connection with behavior, *learning* has also emerged as a significant GO term. Peripheral interactions further divided this cluster into two smaller, but still interpretable clusters (Fig. 6c). The first one (CC3:FC3) includes the neuronal-specific processes such as *ion transport, ion homeostasis regulation of membrane potential*, and *synaptic signaling*, while the second subcluster (CC3:FC4) connects genes with general cell interaction processes such as *cell–cell adhesion*.

The fourth cluster (CC4) is the smallest (100 genes), the biological processes are enriched, but they lost their significance after multiple hypothesis correction. Most of the genes in this cluster have *cell motion* (25 genes), *angiogenesis* (6 genes), and blood vessel-related functions. Subdividing increases the interpretability of the subclusters, but among the resulting three enriched subclusters only one had GO terms with significant *q*-values (CC4:FC1) such as *regulation of blood coagulation* (*p*-value: 4.76 × 10^−7^, *q*-value: 0.00162, hypergeometric test) (Fig. 6d).

**Fig. 7:**
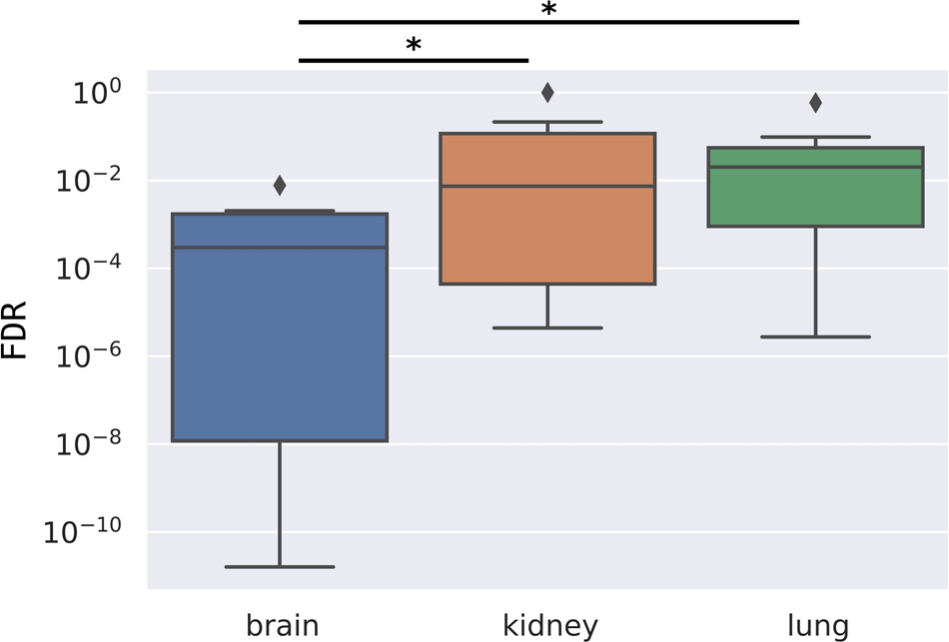
Autism-related GO terms enriched more significantly in the brain subclusters than in kidney and lung. Q-values (FDR) of the autism-related GO terms are presented on the box plot; brain with a blue, kidney with a red, and lung with a green box (centerline, median; box limits, upper and lower quartiles; whiskers, 1.5x interquartile range; diamonds, outliers). Statistical test: Mann–Whitney U test, significant differences are marked by *(*p* < 0.05).

Tissue specificity of the functional results was tested by analyzing the clusters of kidney and lung networks. The autism-related GO terms from Fig. 6 were more significantly enriched in the brain clusters than in the other tissues, where they were less significantly enriched or not enriched at all (*p*: 2.312 × 10^−2^ for brain vs. kidney; *p*: 9.647 × 10^−3^ for brain vs. lung, Mann–Whitney U test) (Fig. 7).

### Functionally interpretable clusters are connected to co-morbidity between autism and intellectual disability

A major implication of the omnigenic theory is that core genes have a direct effect on the manifestation of a disease. Therefore, we hypothesized that functionally different subclusters of core genes would associate with different subtypes of autism.

To investigate this theory, we reanalyzed a large-scale genetic dataset of autism from Satterstrom et al.^4^ This dataset contains a few phenotypic properties of individuals with rare/de novo variants. Among the ASD subjects 3244 also had both IQ and ‘Age of Walking’ data.

First, IQ and age of walking values of individuals with variants in any of the SFARI genes (SFARI group) were compared to the individuals without SFARI gene affecting variants (non-SFARI group). The IQ values of the SFARI group (mean: 76.781) were significantly lower than the non-SFARI group (mean: 82.225) by 5.444 points (*p*: 9.411 × 10^−8^, df: 1722.9, 95% CI [−7.436 −3.452], two-sided, two-sample *t*-test). The age of walking values also showed a significant difference (0.764 month, *p*: 1.168 × 10^−5^, df: 1470.8, 95% CI [0.423, 1.105], two-sided, two-sample *t*-test): individuals in the SFARI group had delayed age of walking (mean: 14.529) compared to the non-SFARI group (mean: 13.765).

Individuals were grouped based on their association with core subclusters in order to study whether the peripheral influence-based clustering can identify phenotypically different subgroups inside the SFARI group. After correcting for multiple testing (FDR, *n* = 14), only individuals with variants in Neuronal Communication I (CC3:FC3) genes had a significantly lower IQ score (mean: 73.327) than that of individuals without mutations in that subcluster (mean: 81.407) by 8.08 points (FDR: 3.002 × 10^−5^, df: 348.44, 95% CI [4.783, 11.376], two-sided, two-sample *t*-test).

By studying ‘age of walking’ values, another group of ASD subjects could be defined. Carriers of disruptive variants in *Gene Expression Regulation II* (CC2:FC2) genes showed significantly delayed age of walking (15.138 months versus 13.874 months) with a difference of 1.264 months (FDR: 7.944 × 10^−4^, df: 285.86, 95% CI [0.655, 1.873], two-sided, two-sample *t*-test).

## Discussion

**Fig. 8:**
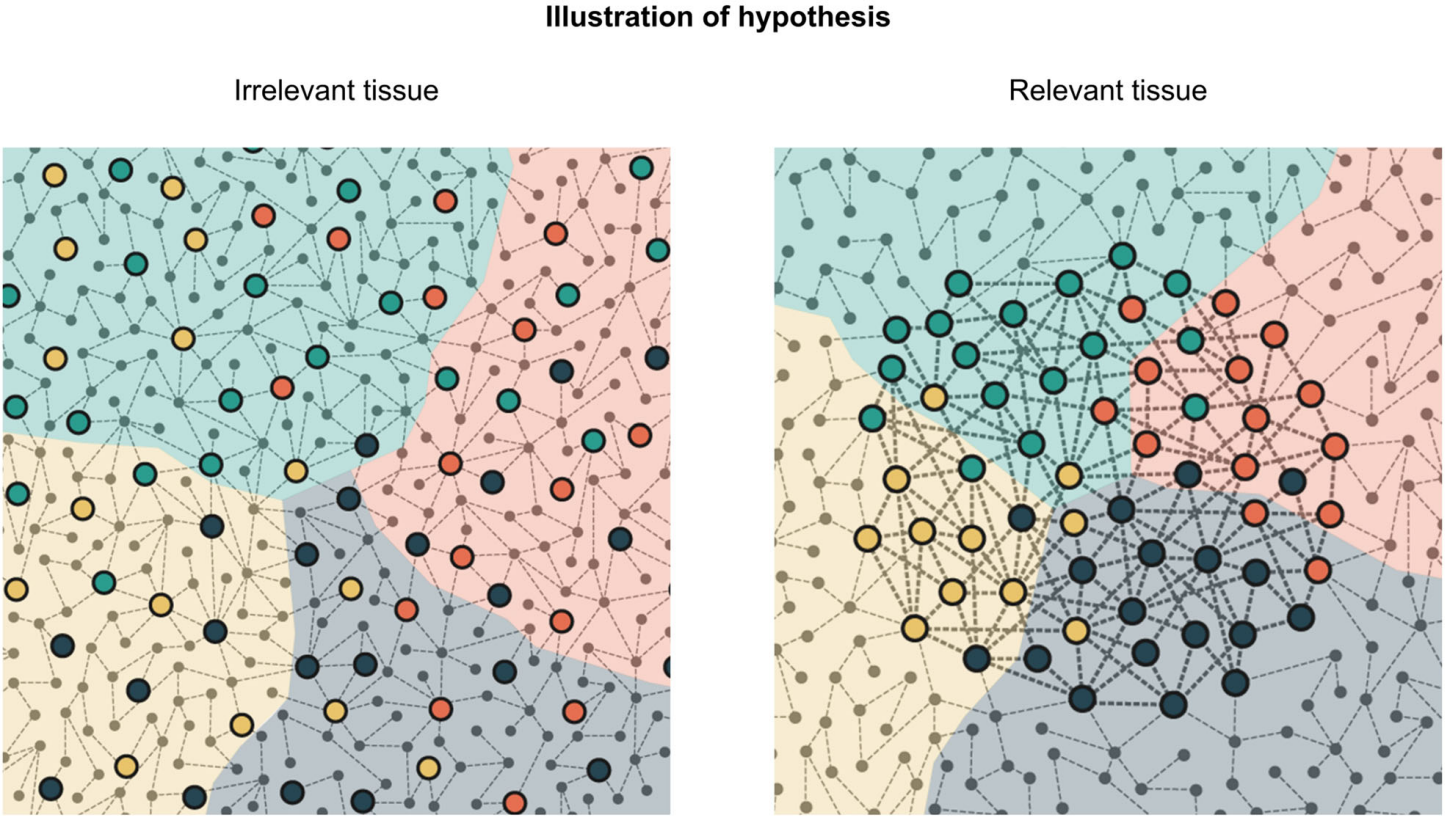
Graphical illustration of the omnigenic theory-inspired clustering. Background colors represent clusters of the entire graph, referred in the text as full clusters. Highlighted nodes are core genes with direct effect. Colors of highlighted nodes indicate cluster assignments on the core subgraph. On an irrelevant tissue, core genes are more scattered with weaker connections to each other. On relevant tissues, core clusters are stronger and overlap with the full clusters.

The aim of this study is to exploit tissue-specific gene interaction networks to endorse the tissue specificity of the omnigenic model and find autism gene clusters with distinct functional properties. The omnigenic hypothesis suggests that small perturbations caused by peripheral genes are propagated toward core genes and have an aggregate influence on phenotype. One possible way to extract this effect is to study gene interactions organized in graph structures. The importance of tissue specificity is a key element of the hypothesis, as the core/peripheral gene distinction is plausible only in tissues relevant to the disorder. In the brain, among the putative autism genes, there is a high number of core genes from both the omnigenic and the network perspectives. These genes have higher node strength in the brain-related GIANT networks than in the networks without any association to the brain. In the case of autism, clustering gene interactions should therefore give us an overall picture of core and peripheral gene community structures in the brain tissue. Clusters of the putative core genes were previously analyzed, and these studies gave insights into the direct genetic contributors to the disorder. However, the two types of clusters (core and full network) can be studied together by analyzing their interrelations. Peripheral influence from certain full network clusters toward core clusters can reinforce or weaken the distinction between the core clusters. Cramer’s V quantifies this relationship by measuring the dependence between the community structure of the core subgraph and the full network. Combining this concept with our motivation to verify the relevance of tissue-specific gene interactions, our work shows that on a relevant tissue peripheral influence is more concentrated on core gene clusters, while on irrelevant ones it is more scattered. The illustrative drawing in Fig. 8 provides a graphical overview of this concept.

By studying the core and peripheral community structures of tissue-specific networks, we could also support new evidence for the tissue specificity of the omnigenic model. Peripheral and putative core genes of autism formed the most similar community structures in the most relevant tissue to autism: the brain. It suggests that peripheral influences converge to distinct clusters of autism genes.

Our functional analysis also emphasizes the importance of peripheral influences. The theoretical consideration behind the functional evaluation of clustering results is that strongly connected genes more probably have similar functions than loosely connected ones. This observation is referred to as ‘guilt by association’^23^, which is a commonly used approach to predict the function of genes with unknown biological role^24^. Since autism core genes are strongly connected in brain tissues, the functional description of the brain clusters is more plausible than their clustering on non-neuronal tissues. This is further strengthened by the fact that the most important biological functions are generally more significantly enriched in the brain. Random rearrangements of the SFARI genes showed that GIANT-based clustering detected communities with high functional homogeneities in the brain.

Most of these biological processes are consistent with previous functional studies of autism genes, such as *synaptic signaling* since communication problems between neurons are considered to be one of the main causes of autism^25^.

Another large subcluster contains genes related to *epigenetic regulation*. The structure of chromatin and especially histones play an important role in the regulation of gene expression and thus also in the amount of protein formed^26^. These functions are also frequently associated with autism^27^.

A further large subcluster is related to *intracellular signaling*, which includes important autism genes such as mTOR being a central component of cell growth^28^. Processes affecting cell life, such as cell growth or cell division, in certain forms of autism, such as comorbid autism with increased head diameter, certainly play a role^29^. Problems with brain development can in some cases also be used as diagnostic markers^30^ and although studies focus more on the formation of neurons, our results suggest that the formation of blood vessels in the brain could be also important since they play a crucial role in brain development and function^31^.

Further processes have also emerged, such as *learning*, specific *post-translational modification of proteins* such as *ubiquitination*, or *blood vessel formation*, which have so far only been linked to the genetic background of autism in a targeted immunocytochemical study and in syndromic form of ASD^32,33^. *Regulation of lymphocyte activation* and *regulation of lymphocyte proliferation* are also two interesting functions since perturbed gene networks of leukocytes have been connected to ASD^34^. Lymphocytes are key factors in immune function, inflammatory responses to infections, and cross-reactions between the fetal and maternal immune systems, which also arise as possible causes of autism^35^.

Based on these analyses, clustering putative core genes of autism on brain networks results in functionally more distinct groups than on other tissues. The same clusters were detected both on the subnetwork of SFARI genes and the full brain network when peripheral interactions were also considered. The functions of brain clusters are correlated well with previous findings on autism, but the clusters of other tissues are not. These analyses also identified the brain subclusters in which the putative core genes are more probably “real” core genes from an omnigenic perspective. In five of six functionally interpretable subclusters, regulation-related or neuronal-specific terms were also enriched (see Fig. 6). The only exception is the *Neuronal Communication II* subcluster, which is formed by intersecting the *Neuronal Communication* core cluster (CC3) with the fourth full cluster (FC4). In this subcluster, only general cell–cell adhesion-related terms were enriched without any regulatory function. Hence the direct effect of these genes on autism pathobiology could not be identified, thus their core gene nature could not be supported. Furthermore, SFARI genes in the functionally unlabeled subclusters (CC1:FC1-3, CC2:FC3-4, CC3:FC1-2, CC4:FC2-4) should not be considered core genes either.

Our research also sheds light on biological processes that may be points of contact in the joint study of genetic and phenotypic data. Indeed, ASD subjects with disruptive mutations in the *Neuronal Communication I* subcluster have significantly lower IQ than other disruptive variants carrying ASD individuals. Furthermore, delayed age of walking was associated with mutations in the *Gene Expression Regulation II* subcluster.

The altered IQ and Age of Walking values of the SFARI group correlate well with the original results of Satterstrom et al.^4^. Despite they grouped the ASD individuals based on different sets of ASD genes, we found similar tendencies.

An advantage of our peripheral influence-based clustering is that it identified a unique subgroup of individuals with lower IQ and a separate one with delayed age of walking. These results demonstrate that refinement of core gene clustering could be beneficial for patient stratification and the design of therapeutical treatments. By utilizing the patients’ genetic variants, therapies could be connected to functionally comprehensible and distinct clusters of genes. This way the patients’ autism-causing genetic background could be described by their unique combinations of the autism gene clusters and may open the route to the proper medication for patients affected by altered single clusters or cluster combinations.

Besides the benefits of our approach, it also has its limitations. Despite we could identify functionally different subclusters of the SFARI genes, the capability of our method to find new ASD functions is limited. Finding clusters of ASD genes that are associated with more specific functions would need a finer resolution clustering. Another limitation of the study is that gene interaction networks do not capture the dynamic nature of gene interactions being important for an ideal formulation of the omnigenic model. Intersecting the core clusters with the full clusters also ignores the temporal changes in the biological systems. These limitations may be overcome by collecting data in longitudinal studies. Considerable amount of information could be collected from such studies in the case of complex disorders such as autism.

## Methods

### Gene interaction networks

#### Full (GIANT) network

The GIANT network was downloaded from HumanBase (https://hb.flatironinstitute.org) website. ‘Top Edges’ network type. Network data containing gene ID pairs (entrez) and weights was read into a pandas dataframe and subsequently used to construct a weighted NetworkX^36^ graph.

#### Core (SFARI) subgraph

Core genes were selected from the SFARI^37^ database (Q2 2019 release), based on gene scores that represent the strength of their association with ASD (genes with gene scores 5 and 6 were excluded). ASD subgraphs for each tissue were constructed keeping only those edges that connect two SFARI genes. As the networks are tissue-specific, the size of intersection with SFARI genes (therefore the size of the resulting SFARI subgraph) is also different in each tissue. SFARI genes are available with HGNC symbol ID, while GIANT^17^ nodes are with entrez IDs. To make them compatible, SFARI gene ids were converted to HGNC using biomart^38^ queries.

#### Graph sampling

For each tissue, the number of selected nodes is proportional to the number of SFARI genes in that tissue. The probability of node selection is weighted by the node strength values of the original network. Only those edges were kept that connected the sampled nodes. Graph sampling was repeated 10 times.

### Clustering

Once the network is prepared as a NetworkX weighted graph, Louvain clustering algorithm^19^ from python-louvain (https://pypi.org/project/python-louvain/) package is used.

As the clustering result from the Louvain algorithm is non-deterministic, the final form of the brain contingency table is created by iterating through core and full clusters and then reordering the columns to have larger numbers close to diagonal.

### Node strength

Node strengths were calculated by Unix and R scripts and from the ‘degree’ property of the NetworkX graph object, with the weight parameter specified to sum up adjacent weights (since we worked with weighted graphs, strength was calculated based on weights instead of degree, which is based on the number of edges), which returns a DegreeView object containing the strength values for each node.

### Measuring dependence with Cramers’ V

Once a contingency table is obtained, Chi-squared statistics is calculated as follows:

$$X^2 = \mathop {\sum}\limits_{i,j} {\frac{{\left( {n_{i,j} - \frac{{n_{i.}n_{.j}}}{n}} \right)^2}}{{\frac{{n_{i.}n_{.j}}}{n}}}}$$

To normalize across contingency tables with different sizes, we calculated Cramer’s V

$$V = \sqrt {\frac{{{\varphi}^2}}{{\min \left( {k - 1,r - 1} \right)}}} = \sqrt {\frac{{x^2/n}}{{\min \left({k - 1,r - 1} \right)}}}$$

where *X*^2^ is the result of Chi-squared test, *n* is the number of observations, *k* is the number of columns, *r* is the number of rows.

These equations are implemented in the NumPy^39^ python package.

### Benchmark network

The benchmark network was generated by the extended version of the Lancichinetti–Fortunato–Radicchi (LFR) algorithm (https://github.com/eXascaleInfolab/LFR-Benchmark_UndirWeightOvp)^40^ with the following parameters: -N 25000, -k 300, -maxk 3000, -t1 2, -t2 1.5, -muw 0.2, -minc 4000, -maxc 8000.

### Statistical tests

Mann–Whitney U test (for node strength differences) was calculated using scikit-learn^41^ python package. T-tests were calculated by t.test R function^42^. The 95% Confidence Intervals are given as 95% CI [Lower limit of confidence interval, Upper limit of confidence interval]. On the figures, * marks significance with alfa = 0.05.

### Gene Ontology analysis

For functional Gene Ontology^43^ analysis, we used GOrilla (Gene Ontology enRIchment anaLysis and visuaLizAtion tool)^22^ webtool, with default parameters (*p* < 0.001). All cases, the background was set to all SFARI genes, while targets were selected as either complete clusters or subclusters (based on different peripheral intersections). We selected GO terms with significant FDR-controlled *q*-values (*q* < 0.05).

The number of enriched genes was determined by a unique count of genes from the Gene Ontology Enrichment Analysis output list.

### Phenotypic analysis

The relations between disruptive mutations and phenotypic properties of ASD subjects were studied on the dataset of Satterstrom et al.^4^. Individuals with both IQ and Age of walking data were kept. They were assigned to the subclusters according to their disruptive mutations. Individuals having multiple mutations, both in the tested subcluster and outside that cluster, were assigned exclusively to the tested subcluster. Two-sample *t*-tests compared phenotypic values with alfa cut-off values of 0.05. Multiple comparison correction was performed by False discovery rate controlling method.

## Supplementary information

Supplementary Data 1

## Data Availability

The datasets generated and analyzed for this study are available from the corresponding author upon request.

## Code availability

Codes are freely available at https://github.com/peripheralgeneclusters/.
